# Liquid biopsy in TNBC: significance in diagnostics, prediction, and treatment monitoring

**DOI:** 10.3389/fonc.2025.1607960

**Published:** 2025-08-04

**Authors:** Jiayi Sheng, Xiaolong Zong

**Affiliations:** ^1^ School of Laboratory Medicine and Biotechnology, Southern Medical University, Guangzhou, Guangdong, China; ^2^ Clinical Laboratory, Second Hospital of Tianjin Medical University, Tianjin, China

**Keywords:** TNBC, diagnostics, liquid biopsy, CTCs, CtDNA, miRNAs, lncRNAs

## Abstract

Breast cancer is the most common malignant tumor and the leading cause of mortality among women worldwide. Triple-negative breast cancer (TNBC) is recognized as t the most aggressive form of breast cancer, with a poor prognosis and a high mortality rate within two years. The role of tumor markers in facilitating the early diagnosis, treatment, and monitoring of therapeutic efficacy and prognosis in TNBC is well-established. Currently, tissue biopsy remains the standard clinical method for determining tumor histology and staging. However, the invasive nature of tissue biopsy often leads to poor patient compliance, especially when repeated biopsies are required. In contrast, liquid biopsy offers several advantages: it is non-invasive, sample collection is straightforward, it can reflect the overall tumor burden and heterogeneity, and it allows for real-time monitoring. The markers primarily encompass circulating tumor cells (CTCs), circulating tumor DNA (ctDNA), microRNAs (miRNAs), long ncRNA (lncRNAs), exosome, and so forth. The present review aims to provide a comprehensive overview of the recent advancements and potential clinical applications of liquid biopsy technology in the context of TNBC.

## 1 Introduction

Breast cancer is one of the most common malignancies and the leading cause of death among women around the world (1). Triple-negative breast cancer(TNBC) is a tumor type characterized by the absence of estrogen receptor (ER), progesterone receptors (PR) and HER2 expression. They account for approximately 12%–17% of patients diagnosed with breast cancer (2, 3). A significant body of research has indicated patients with TNBC are notably younger than those diagnosed with other breast cancer subtypes (4). TNBC is the most aggressive subtype, with a poor prognosis and high mortality rate within two years. Moreover, TNBC is susceptible to central nervous system metastases during the initial and subsequent recurrences. This markedly poor prognosis serves to further reduce the survival rate (5). Neoadjuvant chemotherapy has been shown to increase the pathological complete response (pCR) rate in patients diagnosed with TNBC (6). However, this treatment approach has also been linked to a reduction in both three-year progression-free survival (PFS) and three-year survival rates in individuals with this specific form of breast cancer (7). In patients with advanced TNBC, the immunotherapy PD-1 inhibitor pembrolizumab has no significant therapeutic effect (8). Similarly, the use of carboplatin and docetaxel did not improve efficacy or overall survival in patients with metastatic or recurrent locally advanced TNBC (9).It can be reasonably deduced that the implementation of early diagnosis and treatment protocols represents an efficacious strategy for the reduction of mortality rates among patients diagnosed with primary breast cancer.

A clinical diagnosis requires tissue biopsy to ascertain the tumor’s histology and stage. Nevertheless, there are certain constraints and potential complications associated with the surgical procedure of collecting tissue biopsies. Additionally, this technique is limited by the spatial and temporal heterogeneity of tumors. For patients with solid tumors such as TNBC, the presence of microscopic metastatic lesions that cannot be detected by current imaging modalities, such as computed tomography (CT) or positron emission tomography (PET) significantly complicates the prevention and treatment of fully metastatic disease (10). Liquid biopsy, defined as a noninvasive method that detects cancer-related biomarkers in blood or other body fluids, represents a promising approach for the early detection of cancer in asymptomatic individuals and those at high risk, with the potential to be integrated into screening programs utilizing non-invasive methods based on the analysis of blood or body secretions (11). This may result in the earlier detection of cancer, which could in turn lead to improved cure and survival rates. For patients with confirmed tumors, liquid biopsy offers a straightforward method of obtaining blood samples and monitoring tumors in real time (12).

The field of liquid biopsy has witnessed significant advancements, evolving from the initial detection of circulating tumor cells (CTCs) (13) and circulating tumor DNA (ctDNA) (14) to encompass the detection of exosomes (15), microRNAs (miRNAs), peripheral blood circulating RNA, tumor-educated platelets (TEPs) (16) and circulating tumor vascular endothelial cells (CTECs) (17). The heterogeneity of TNBC patients places high demands on detection technology. Currently, TNBC markers can be captured and detected more sensitively using custom personalized ctDNA microarrays (18), liquid chromatography-tandem mass spectrometry (LC-MS/MS) lipid profiling (19), the c-TRAK TN trial (20), miRNA single-molecule sensors (21), near-infrared (NIR) probes (22) and other technologies. In light of the rapid advancements being made in the field of liquid biopsy at present, this review summarizes of the research progresses and clinical applications of liquid biopsy technology in the context of TNBC, including early diagnosis, personalized treatment, prognosis prediction and the advancement of related technologies.

## 2 CTCs

### 2.1 Biological significance of CTCs

The concept of CTCs was first described by an Australian doctor in 1869 during an autopsy on a patient who had died of advanced metastatic cancer (23). CTCs are cancer cells originating from solid tumor which are found circulating in the peripheral blood. It is generally accepted that these cells are shed into the bloodstream from the primary or secondary tumor of patients with advanced cancer, and studies have shown that the morphology of CTCs varies depending on the stage and/or type of tumor (24). Compared to traditional invasive tissue biopsies, CTCs are not only easy to sampled, but also offer real-time insights into the dynamic changes in CTC levels (25). As CTCs can originate from both primary and (micro)metastatic sites, they can better represent the entire heterogeneous tumor cell population (26).

### 2.2 Evolution of CTC detection technologies

In general, CTC detection involves two steps: enrichment or separation—using pathological and immunological techniques—and detection or identification—using cytometry and nucleic acid (27). Early CTC detection techniques based on nucleic acid detection of CTCs included purification of nucleated cells, followed by lysis and nucleic acid extraction (28), and sequencing of individual tumor-specific translocation breakpoints (29). However, this method of nucleic acid analysis from free plasma or unpurified blood cell components has relatively low sensitivity. Moreover, it is difficult to distinguish whether the absence of a clear molecular abnormality in the blood sample is due to the lack of such abnormalities or to an insufficient amount of tumor-derived DNA circulating in the bloodstream of an individual patient (30).

Currently, enrichment methods for CTC detection fall into two broad categories: label-free and affinity-based. Label-free methods for CTC enrichment are achieved by exploiting biophysical properties, as these methods are independent of biomarkers and can capture cells regardless of CTC expression profiles (31). These methods enable the isolation and enrichment of epithelial-mesenchymal transition (EMT) cells, cells with stem-like cancer properties (32), and fibroblasts that may be associated with cancer (33). Immunomagnetic CTC enrichment is the most widely used strategy, which relies on the use of cell surface-expressed biomarkers. Tumor cells are positively selected by anti-epithelial cell adhesion molecule (EpCAM). Alternatively, the leukocyte background is removed by targeting the CD45 antigen expressed on leukocytes, thereby achieving negative selection (31). As the technology evolves, a variety of techniques are available to selectively detect viable CTCs, such as the Epithelial ImmunoSPOT (EPISPOT) assay, which can detect single cells of CTCs and has been successfully used in a large cohorts of breast cancer patients (34). With the development of technology, the technique of isolating CTCs from patients has gradually matured and can provide more accurate information in real-time for early detection, treatment and prognosis of tumors.

### 2.3 Clinical utility of CTCs in TNBC

In patients with aggressive cancers, metastasis can occur at an early stage of tumor development and is the leading cause of cancer-related deaths (35). The invasion of cancer cells into the bloodstream and their spread to other parts of the body via the bloodstream is the first step in metastasis. Studies of disseminated tumor cells (DTCs) in the bone marrow of breast cancer patients have shown that even patients with small early tumors can have early spread to distant sites (36). Therefore, the primary indicator of the early-stage of cancer metastasis may be CTCs in the blood. In early-stage TNBC patients, CTCs were detected in tumors of histological grades 1–4 tumors, with a detection rate of 17.8% for grade 2 tumors and 60% for grade 3 tumors, indicating the high sensitivity of CTC testing in identifying TNBC patients (37). Therefore, CTCs have important value and research prospects for early detection of TNBC.

CTC testing can detect molecular disease recurrence prior to radiographic or clinical manifestations and predict treatment response prior to surgery. This ‘lead time’, allows clinicians to take pre-emptive action earlier in patients who are not responding well to traditional chemotherapy regimens (38). A study of 56 metastatic breast cancer patients (10 of whom had the TNBC subtype) found that the prognosis for PFS was worse in patients with positive CTCs at baseline. Moreover, a favorable prognosis was associated with a ≥70% reduction in CTC count from baseline or fewer than 5 CTCs in 7.5 mL of blood before the second cycle of third-line chemotherapy. These results suggest that early changes in CTCs during systemic therapy may have predictive value for treatment outcome (39). Similarly, Paoletti C. et al. analyzed the number of CTCs, the presence of CTC clusters and the dynamics of apoptotic CTCs in 64 mTNBC patients prior to chemotherapy with nanoparticle albumin-bound paclitaxel or chemotherapy without the addition of trastuzumab. This study showed that a reduction in the number of CTCs was associated with improved PFS in patients and indicated a response to treatment (40). Furthermore, longitudinal CTC assessment by Magbanua et al. revealed that post-treatment CTC counts (days 7-14) better predicted progression risk than baseline values in TNBC patients (41). Consequently, CTC represents a promising tumor marker that can be readily obtained from peripheral blood. It has the potential to become a valuable tool in the future, enabling the refinement of treatment plans for tumors.

Besides its established role in predicting treatment response, accumulating evidence confirms CTCs’ prognostic value in TNBC. Peeters D.J.E. et al. demonstrated no significant difference in CTC counts across breast cancer subtypes, The survival rate and prognosis of breast cancer patients with a CTC count of 5 or more in a 7.5 ml blood sample is significantly worse than that of patients with a CTC count of less than 5 (42). Tsai et al. corroborated this threshold, finding <5 CTCs conferred only 12.5% distant metastasis risk versus ≥5 CTCs (43). Importantly, Jansson et al. reported TNBC patients with CTC clusters within 3 months post-diagnosis exhibited significantly shorter PFS/OS versus cluster-negative cases, though this difference attenuated by 6 months (44). The ability of CTCs to predict the prognosis of TNBC has shown good results, allowing physicians to adjust treatment plans for TNBC patients to prolong their survival. The use of CTCs as a tumor marker, which can be readily obtained from peripheral blood, may become more prevalent in the future, particularly in guiding the diagnosis and treatment of tumors.

### 2.4 Integrated single-cell omics for CTC characterization

Single-cell technologies provide unprecedented resolution for profiling circulating tumor cells (CTCs) in breast cancer, enabling deep insights into tumor heterogeneity and metastasis mechanisms. Single-cell RNA sequencing (scRNA-seq) deciphers gene expression at cellular resolution, distinguishing breast cancer subtypes and identifying subpopulations linked to poor prognosis and drug resistance (45). Integrated genomic/epigenomic analyses track clonal evolution and genetic instability driving metastatic spread (46). Additionally, single-cell proteomics maps protein networks underlying functional heterogeneity and therapy resistance. These approaches synergistically enhance CTC detection sensitivity. CTC quantities and molecular features (e.g., EMT phenotypes) correlate with early metastasis risk and survival outcomes (47). Clinically, scRNA-seq of CTCs identifies resistant subpopulations, enabling real-time therapy adjustments, while CTC genomic profiles guide personalized regimens. Moving forward, enhancing detection specificity and advancing multi-omics integration will maximize clinical translation.

## 3 ctDNA

### 3.1 Biological origins of ctDNA

The existence of tumor-derived cell-free DNA (cfDNA) in the blood was proposed by Stroun et al. in 1989 (47). ctDNA is a type of cfDNA shed by apoptotic or necrotic tumor cells and is derived from the tumor cells themselves (48). Tumor cells consist of malignant cells and cells of the tumor microenvironment, such as stromal cells, endothelial cells, lymphocytes and other immune cells. All of these cells can be a potential source of ctDNA (49). ctDNA can even originate from CTCs in the blood (50). ctDNA fragments are predominantly double-stranded with a length of approximately 132–145 base pairs (51). They have a short half-life of approximately 2 hours. In addition, the tumor–molecular complex binding or the type and stage of the tumor, treatment and other factors can affect the amount and nature of ctDNA (52). ctDNA can collect tumor fragments from multiple lesions in the body, with less spatial variation than needle biopsy, and can better capture heterogeneity (53). Due to these characteristics, ctDNA has the potential to reflect the occurrence and evolution of tumors in the body in real time. The real-time detection of ctDNA has been the subject of considerable interest and has established ctDNA as a promising tumor marker.

### 3.2 Evolution of ctDNA detection technologies

ctDNA fragments are predominantly double-stranded fragments of approximately 132–145 base pairs with a short half-life of approximately 2 hours (54). In cancer patients, 1 mL of plasma contains approximately 1,500 diploid genome equivalents (GE), equivalent to ~10 ng of DNA. From 10 mL of blood, roughly 4 mL of plasma can be extracted, yielding about 6,000 GE. This sets a theoretical sensitivity limit of 0.1% for mutation detection (54), posing significant challenges for developing reliable assays. Digital PCR (dPCR) addresses this limitation. Pioneered by Vogelstein & Kinzler (55), it transforms exponential PCR amplification into linear quantification. The method partitions DNA into microdroplets for endpoint amplification, with target concentration calculated from ratios of fluorescence-positive droplets. Next-generation sequencing (NGS) offers distinct advantages as a non-targeted approach, detecting emerging alterations—including copy number variants, structural rearrangements, and single nucleotide substitutions. However, NGS faces sensitivity constraints: its typical 30-100x coverage in whole exome/genome sequencing struggles to identify mutations below 5% allele frequency (56). Although the non-targeted approach is less sensitive than the targeted approach, one of its potential advantages is that it can detect subclonal mutations that are less common in the primary tumor but selected during adjuvant therapy or the natural disease course after surgery (57).

### 3.3 Clinical utility of ctDNA in TNBC management

Currently, neoadjuvant chemotherapy remains the primary systemic pharmacological intervention for patients with TNBC. Given the favorable treatment response observed in patients with early-stage TNBC, it is of paramount importance to implement effective strategies for the early detection of this disease (58). A comparable study conducted by Olsson and colleagues demonstrated that serial ctDNA monitoring in patients with primary breast cancer can yield an average lead time of up to 11 months before the onset of metastatic disease (59). Garcia-Murillas et al. conducted a prospective study in 55 high-risk early-stage breast cancer patients treated with neoadjuvant chemotherapy and surgery. By designing patient-specific ddPCR assays for tumor mutations, they achieved an 80% sensitivity for relapse prediction on (60). In a larger multicenter cohort of 101 breast cancer patients (including TNBC), periodic ddPCR monitoring revealed that ctDNA detection during follow-up was strongly associated with relapse and preceded clinical relapse by a median of 10.7 months. Together, these studies underscore ctDNA’s ability to reveal minimal residual disease (MRD) and pre-empt clinical recurrence by many months, creating a potential window for therapeutic intervention (61).

Additionally, ctDNA represents a valuable tool for predicting the treatment effect, recurrence rate, and survival rate of TNBC patients. In metastatic TNBC, ctDNA demonstrates 81% sensitivity and 97% concordance with tissue biopsy for mutation profiling (62). Consequently, ctDNA testing can be employed as a non-invasive and real-time method for the detection of metastatic TNBC progression, thereby facilitating the timely adjustment of treatment plans. Furthermore, a study conducted by Francesca Riva and colleagues on 46 patients with non-metastatic TNBC demonstrated a significant correlation between ctDNA levels and several clinical parameters, including continuous variation, mitotic index, tumor grade and tumor stage. It is thus feasible to more accurately monitor the patient’s treatment response in real time during neoadjuvant chemotherapy (NCT). Furthermore, this method is also applicable to postoperative treatment (63). In a clinical study of 196 early TNBC patients, Milan Radovich and colleagues observed a significant decline in distant disease-free survival (DDFS) among patients with ctDNA detected in the blood following neoadjuvant chemotherapy. The 24-month DDFS rate for patients with ctDNA positivity was 56%, while that for patients with ctDNA negativity was as high as 81%. A similar trend was observed with respect to disease-free survival (DFS) and overall survival (OS) (64). Residual cancer burden (RCB) stratification further reveals escalating ctDNA positivity rates (RCB-I:14%, RCB-II:31%, RCB-III:57%) coupled with declining 3-year event-free survival (EFS: 100%/79%/23%) (65). Furthermore, digital pCR mutation tracking demonstrated that the median recurrence time for TNBC is 10.6 months, indicating a high propensity for recurrence in the short term (66). Consequently, the real-time detection of ctDNA enables the implementation of timely interventions to enhance the prognosis and prolong the survival period in instances where ctDNA is identified, showing the potential to link disease progression to one quantitative and non-invasive method in TNBC.

However, clonal hematopoiesis of indeterminate potential (CHIP) confounds cfDNA-based liquid biopsy analysis, as somatic mutations in genes including DNMT3A, TET2, ASXL1, PPM1D, and TP53 from age-associated hematopoietic clones can mimic tumor-derived variants (67). In breast cancer cohorts, ≈15% of patients harbor CHIP mutations at diagnosis, with chemotherapy promoting expansion of TP53-mutant clones (variant allele frequency ≥0.5%). Metastatic triple-negative breast cancer (TNBC) exhibits comparable CHIP prevalence without demonstrable survival impact, necessitating rigorous discrimination between leukocyte-derived DNA and true tumor ctDNA (68). Effective mitigation combines three core strategies: concurrent sequencing of matched peripheral blood leukocytes, application of <5% variant allele frequency (VAF) thresholds for CHIP exclusion, and bioinformatic classifiers trained on mutational signatures, collectively enhancing ctDNA analysis specificity for clinical deployment.

## 4 miRNAs

### 4.1 Biogenesis and functions of miRNAs

It is estimated that approximately 75% of the human genes are transcribed into RNA, of which only about 3% are subsequently translated into proteins (69). Non-coding RNAs (ncRNAs) are classified by their length, structure, and subcellular localization. The most notable types of ncRNA include miRNA, long ncRNA (lncRNA), circular RNA (circRNA) and PIWI-interacting RNA (piRNA); each has distinct functions in cancer (70). miRNA is a type of small non-coding RNA (sncRNA) that is approximately 22 nucleotides in length. It has the capacity to influence the progression of tumors by post-transcriptionally targeting oncogenes or tumor suppressors (71). It has been demonstrated that the upregulation of miRNA has a role in the promotion of carcinogenesis, while the downregulation of certain miRNA has been identified as a potential antitumor agent. Aberrant expression of these molecules has been linked to the regulation of critical biological processes, including the ABC transporter, cell death, cell cycle, and signaling pathways, which are associated with tumor development, including the progression, metastasis, and chemoresistance of TNBC (72).

### 4.2 Technical challenges in miRNA detection

miRNA is characterized by their short length and natural occurrence at low abundance in biological samples, which necessitates the employment of highly sensitive detection methods. Reverse transcription-quantitative PCR (RT-qPCR) is the gold standard for miRNA quantification (73). RT-qPCR is characterized by its high sensitivity and specificity, and is widely regarded as the gold standard for the detection of miRNAs (74). However, RT-qPCR analysis of miRNAs necessitates a multitude of sample preparation steps and the utilization of various enzymes, thereby augmenting the cost and complexity of the analysis (75). Currently, next-generation sequencing (NGS) and DNA microarrays are also available for the detection of miRNAs. NGS has the capacity to detect multiple targets, a property that is valuable for the discovery of novel miRNAs and comprehensive analysis. Nevertheless, due to the time-consuming and costly nature of next-generation sequencing technology, its use in rapid clinical testing and large-scale applications in early diagnosis is not feasible (76). DNA microarrays are extensively utilized for the detection and diagnosis of miRNAs, as well as target selection. The advantages of DNA microarrays include their relatively low cost, high throughput, and the capacity for multiplex detection. Nevertheless, traditional DNA microarrays are constrained by protracted detection times and intricate operating procedures, encompassing PCR amplification, target labelling and washing steps. This is particularly challenging for miRNAs, a type of short-chain RNA, due to the complex and time-consuming nature of amplification and fluorescent labelling (77). Chen et al. established a fluorescent detection method based on SCas12a RNP, which has been demonstrated to be capable of detecting miRNAs with high sensitivity, high selectivity and multiplexing, without reverse transcription or preamplification (78). Haruka et al. established a signal probe-based hybridization system for simple PCR, target and cleaning-free detection of miRNAs. The signal probe has been demonstrated to be capable of detecting a variety of different miRNAs, encompassing those of varying lengths and GC contents, including those with high GC content. The detection method has the potential for use in liquid biopsy and is expected to achieve economical, high-throughput and multiplex detection of miRNAs (79).

### 4.3 miRNA signatures in TNBC diagnosis

As a result of the application of high-throughput sequencing technology, the number of mature miRNAs discovered has now exceeded 28,000. This represents between 1% and 5% of the human genome. Kumar et al. conducted a study in which the expression of microRNAs was analyzed based on qRT-PCR. The results of this study demonstrated that the expression levels of miR-155 and miR-21 were significantly elevated in patients diagnosed with TNBC, while the expression levels of miRNA-205 were markedly reduced. The disruption of these miRNA expressions can thus be used as an independent indicator to distinguish TNBC from healthy patients (80). Kalani et al. developed a HJ and FRET-based single-molecule sensor capable of detecting TNBC-related miRNAs (miR-342-3p) with high sensitivity and specificity. It is anticipated that this sensor will serve as a novel approach for the early diagnosis of TNBC through the analysis of body fluids (21).

In comparison with other tissues, the high expression of miRNA in TNBC tissue can be utilized as a reference for the diagnosis of TNBC. Hou et al. found that the expression of miRNA-1207-5p was significantly increased in tissue samples of TNBC compared to adjacent normal tissue. Furthermore, they found that the expression of miR-1207-5p was significantly higher in TNBC tissue non-responsive to Taxol compared to Taxol-responsive (81). It is hypothesized that miR-1207-5p has the potential to serve as a novel biomarker for the diagnosis of TNBC.

### 4.4 Prognostic and therapeutic potential

miRNA has been demonstrated to be associated with the malignant characteristics of TNBC, including proliferation, migration, metastasis and drug resistance. Telomeric repeat-binding factor 2 (TRF2) has been observed to be overexpressed in a variety of human cancers, including breast cancer, and has been demonstrated to promote cancer cell escape (82). Dinami et al. found that the ectopic expression of miR-182-3p significantly reduced levels of the TRF2 protein, thereby activating DNA damage. Therefore, measuring miR-182-3p levels can serve as an indicator to assess apoptosis in TNBC cells. Furthermore, lipid nanoparticles (LNPs) containing miR-182-3p (LNPs-miR-182-3p) has been demonstrated to facilitate the crossing of the blood-brain barrier, resulting in the reduction of intracranial tumors. These finding offers novel therapeutic prospects for the management of metastatic brain disease (83).

Differential miRNA expression patterns stratify TNBC progression and treatment response. Berber et al. identified suppressed miR-200c and miR-205 as predictors of lymph node metastasis (84), while Song et al. associated miR-1-3p and miR-133a-3p with overall prognosis (85). For chemotherapy response, Li et al. identified miR-770 overexpression as a key sensitivity indicator through transcriptome analysis—a role further validated in TCGA datasets for prognostic stratification (86). Survival-linked miRNAs further refine risk assessment: Deng et al. reported extended median survival (77 vs. 70 months) with high miR-221-3p expression, independent of clinicopathological variables (87), and Svoboda et al. demonstrated negative correlations between miR-34b levels and disease-free/overall survival (88). It is evident from the aforementioned markers that miRNA has the potential to serve as valuable prognostic markers for TNBC. It has been established that a significant proportion of miRNAs are expressed at elevated levels in TNBC, with these molecules demonstrating a strong correlation with the malignant characteristics of tumors. Consequently, these miRNAs have the potential to serve as biomarkers for liquid biopsy TNBC.

## 5 lncRNAs

### 5.1 Biological roles of lncRNAs in TNBC

lncRNA constitute a subclass of non-coding ribonucleic acid (ncRNA) characterized by a sequence length of more than 200 nucleotides and little or no protein-coding potential (89). LncRNA has been found to be present in the cell membrane or nuclear region, interspersed and overlapping regions of coding and non-coding transcripts. These molecules possess a variety of molecular functions and roles, including acting as scaffolds, decoys, or guides; modulating signaling pathways; organizing chromatin architecture; and responding to developmental or environmental stimuli (90). Aberrant expression levels of lncRNA expression is associated with a multitude of malignant biological processes, including genes, proliferation, angiogenesis, EMT and distant metastasis (91). A plethora of studies have demonstrated that aberrant expression of lncRNA plays a pivotal role in the pathogenesis, progression and metastasis of TNBC. Thus, lncRNAs hold significant promise for TNBC liquid-biopsy applications.

### 5.2 lncRNA in TNBC diagnosis

LncRNAs demonstrate functional correlations with diverse regulatory mechanisms—including transcription factor modulation, epigenetic alterations, and post-translational modifications—alongside interactions with small peptides, collectively underpinning their clinical relevance for early TNBC diagnosis and therapeutic intervention (92). Detection of lncRNAs generally parallels miRNA workflows. Swellam et al. utilized qRT-PCR to detect the expression of X inactive specific transcript (XIST) and nu-clear paraspeckle assembly transcript 1 (NEAT1) in serum samples from breast cancer patients, patients with benign breast lesions and healthy volunteers, respectively. Their findings revealed significantly elevated levels in breast cancer patients versus benign/healthy controls, with further increases in TNBC subgroups, establishing these lncRNAs as diagnostic biomarkers (93). Furthermore, Lv et al. utilized microarray detection to identify 880 upregulated and 784 downregulated lncRNAs in TNBC versus non-TNBC with ROC validation confirming RP11-434D9.1, LINC00052, BC016831, and IGKV as diagnostic indicators (94).

### 5.3 lncRNA in the treatment and prognosis of TNBC

LncRNAs play pivotal roles in regulating TNBC proliferation, apoptosis, and drug resistance (95). Zheng et al. demonstrated that lncRNA MILIP is highly expressed in p53-mutant TNBC cells, where silencing suppresses cell viability and xenograft growth. Mechanistically, MILIP interacts with eukaryotic elongation factor 1 alpha 1 (eEF1a1) to form RNA-RNA duplex with the type II tRNAs tRNALeu and tRNASer through their variable loops. This interaction promotes tRNA binding and protein synthesis. Disruption of this interaction reduces cellular viability (96). Parallel studies revealed MALAT1 upregulation in TNBC tissues (43 paired samples) enhances proliferation, invasion and cell cycle progression via the MALAT1/miR-129-5p axis (97). Wang YF et al. found that HIF1A-AS2 expression was significantly higher in TNBC cell lines than in normal breast epithelial cell lines. The findings indicated that elevated HIF1A-AS2 expression was associated with lymph node metastasis, distant metastasis and poor histological grading in patients with TNBC. Furthermore, silencing HIF1A-AS2 expression led to a significant inhibition of the migration and invasion of TNBC cells (98). Emerging evidence reveals that LINK-A mediates drug resistance through PKA-TRIM71 axis activation, compromising immune checkpoint inhibitor efficacy (99). Complementary research shows dual silencing of HIF1A-AS2 and AK124454 in TNBC cells elevates paclitaxel half-maximal inhibitory concentration (IC50) >2-fold. This IC50 increase directly indicates reduced paclitaxel sensitivity, confirming taxane resistance association. Furthermore, the establishment of a drug resistance prediction model based on these two genes has enabled the development of personalized treatment regimens for patients with TNBC (100). The aforementioned markers facilitate the real-time monitoring of TNBC development and metastasis, with significant implications for the monitoring and adjustment of treatment efficacy.

He et al. found that OSTN-AS1 serves as an immune-related biomarker, while T376626 levels correlate with advanced stages, aggressive molecular subtypes, and poor survival (101). Subsequent analysis confirmed elevated LINK-A predicts reduced relapse-free survival (102). In summary, the detection of lncRNAs in blood samples has become a promising method for monitoring the progression of TNBC and for prognostic prediction, which can be incorporated into the clinical management of patients.

## 6 Other biomarkers

### 6.1 Exosomes

Exosomes are 40–160 nm in diameter and consist of a lipid bilayer. These vesicles are released by most cells and circulate stably in body fluids (103). Exosomes are rich in a variety of biologically active molecules, including nucleic acids, proteins and lipids, which can be transferred from donor to recipient cells, facilitating intracellular information transmission (104). Hoshino et al. demonstrated plasma-derived exosome analysis enables cancer-type identification and tumor-origin determination via proteomics (105). Yu et al. found that acetylated LAP-TGF-β1 enters cells via exosomes, accelerating lung metastasis and spread, thereby establishing exosomal TGF-β1 as a promising therapeutic target for metastasis (106). In the study by Zhao et al., it was established that cationic bovine serum albumin (CBSA) conjugated with siS100A4 and exosome membrane-coated biomimetic nanoparticles (CBSA/siS100A4@Exosome) can significantly inhibit the postoperative metastasis of triple-negative breast cancer (107). Consequently, the delivery of siS100A4 by exosome membrane-coated core-shell nanoparticles is anticipated to emerge as a promising clinical strategy for cancer prevention and treatment.

### 6.2 Circular RNA

Circular RNA (circRNA) is a class of endogenous RNA transcripts that feature covalently closed loop structures. In comparison with linear RNA, circRNA is devoid of 5’ caps or 3’ tails, and is distinguished by its enhanced half-life, elevated evolutionary conservation, and augmented resistance to digestion by RNase R (108). In the study by Wang et al., it was found that the expression of circRNA-CREIT (hsa_circ_0001798) was significantly reduced in breast cancer, particularly in chemo resistant breast cancer cells and the TNBC subtype. Additionally, low expression of circRNA-CREIT was found to be associated with higher grade, increased lymph node metastasis, larger tumor volume, and poorer prognosis in breast cancer patients. Furthermore, circRNA-CREIT has been demonstrated to possess the capability to impede the proliferation and migration of TNBC cells, whilst concomitantly inducing apoptosis (109). It was previously reported that circHIF1A promotes the proliferation and metastasis of TNBC by upregulating NFIB (110). The aforementioned markers indicate a strong association between circRNA and the metastasis and prognosis of TNBC, suggesting its potential as a novel and valuable marker.

### 6.3 Tumor-associated neutrophils

Neutrophils, also referred to as polymorphonuclear cells, are a type of granulocyte belonging to the myeloid lineage. These cells represent the most abundant white blood cell type, and they are the most significant immune cells, comprising 50–70% of white blood cells in adults (111). Neutrophils have been shown to play a role in bacterial infections and are also actively involved in various aspects of breast cancer development, including growth, migration/invasion, angiogenesis and metastasis (112). Tumor-associated neutrophils (TANs) are gaining recognition as a significant functional attribute that may influence the prognosis of breast cancer, particularly in the context of the TNBC subtype (113). Breast cancer patients, including TNBC patients, with CTC-neutrophil clusters have worse progression-free survival than those without clustered CTC. Circulating neutrophils confer higher malignant potential to CTCs (114). Furthermore, Wang et al. discovered that elevated levels of tumor-infiltrating neutrophils (TINs) were associated with advanced histological grade, tumor stage, and the TNBC subtype. Mechanistically, parenchymal TINs promote breast cancer cell migration, invasion, and EMT through TIMP-1 secretion in a CD90-mediated cell-contact dependent manner (115). Consequently, CTC-neutrophil clusters show diagnostic potential, while parenchymal TIN density serves as a validated prognostic indicator for TNBC.

## 7 Conclusion and future perspectives

**Figure 1:**
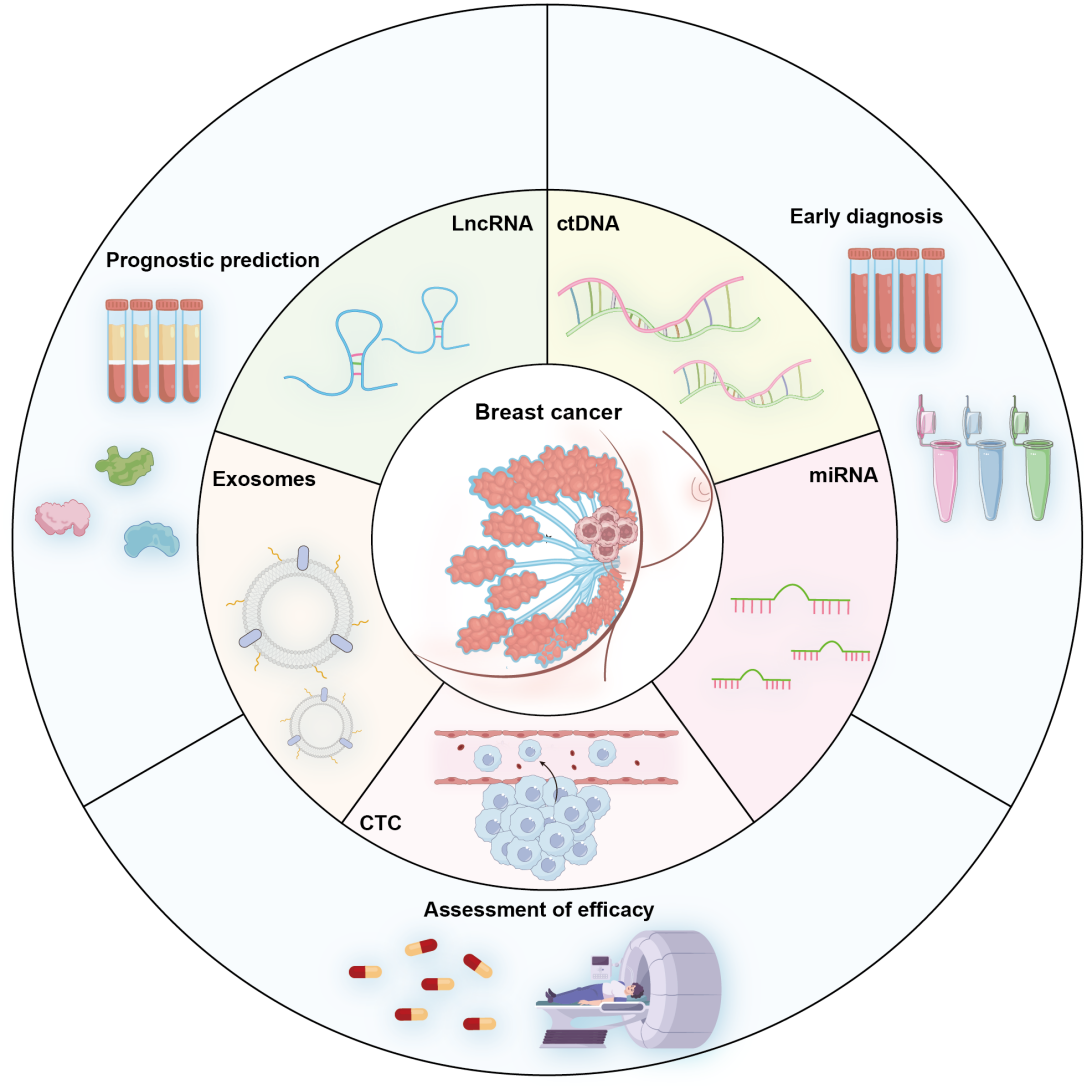
Schematic diagram illustrating key circulating analytes used in TNBC liquid biopsy and their primary clinical utilities including Early diagnosis (screening and detection of initial disease), Prognostic prediction (risk stratification and outcome forecasting), and Evaluation of Treatment Efficacy (monitoring response to therapy and minimal residual disease).

Liquid biopsy represents a paradigm-shifting approach in triple-negative breast cancer (TNBC) management, offering non-invasive access to molecular information through blood/urine biomarkers including CTCs, ctDNA, miRNAs, and lncRNAs (**Figure 1**). This technology enables dynamic monitoring of tumor evolution, facilitating early diagnosis, treatment response assessment, recurrence surveillance, and personalized therapy selection—addressing TNBC’s critical unmet need for targeted therapeutic strategies.

The future development of TNBC liquid biopsy centers on four interconnected priorities. First, integrating multi-analyte signatures through multiplexed detection platforms will enhance diagnostic precision by combining complementary biomarkers into comprehensive panels (**Table 1**). Second, technological breakthroughs in ultrasensitive detection methodologies—such as advanced digital PCR systems, nanopore sequencing, and CRISPR-based diagnostics—promise to overcome current sensitivity limitations by identifying rare variants below 0.1% frequency thresholds, enabling earlier interception of minimal residual disease and pre-symptomatic recurrence.

**Table 1:** Liquid biopsy biomarkers in TNBC: biological characteristics, clinical applications, and technical considerations.

Biomarker	Biological characteristics	Clinical applications	Advantages	Limitations
CTC	- Shed from primary/metastatic tumors - Heterogeneous morphology - Represents tumor heterogeneity - Can enter dormancy	- Early detection: Detected in 17.8–60% of early TNBC tumors (37) - Prognosis: ≥5 CTCs/7.5mL blood predicts poor survival (39) - Therapy monitoring: CTC reduction correlates with improved PFS (40) - Recurrence: Post-treatment CTC counts predict progression (41) - Metastasis: CTC clusters linked to shorter PFS/OS (44)	- Non-invasive - Captures spatial heterogeneity - Enables real-time monitoring - Reflects viable tumor cells	- Low abundance (esp. early-stage) - Technical complexity in isolation - Requires rapid processing
ctDNA	- Fragmented DNA (132–145 bp) - Short half-life (~2 hrs) - Released via apoptosis/necrosis - Pan-tumor origin	- MRD monitoring: Detects residual disease post-NACT (61) - Recurrence prediction: 10–11-month lead time before metastasis (59) - Prognosis: Post-NACT ctDNA linked to DDFS (64, 66) - Treatment resistance: Identifies emerging mutations (62) - Tumor burden: Correlates with RCB grade (62, 65)	- Reflects genomic alterations - High concordance with tissue biopsies (81–97%) (66) - Dynamic tumor monitoring	- Low concentration (sensitivity ~0.1%) (55) - CHIP interference (68, 69) - Requires sensitive tech - Background cfDNA noise
miRNA	- ~22 nt non-coding RNA - Regulates oncogenes/tumor suppressors - Stable in circulation - Dysregulated in TNBC	- Diagnosis: miR-155↑, miR-21↑, miR-205↓ distinguish TNBC (80) - Drug resistance: miR-1207-5p↑ in taxol-resistant TNBC (81) - Prognosis: Low miR-200c/miR-205 predict lymph node metastasis (84) miR-221-3p↑ correlates with longer survival (87) - Therapy response: miR-770↑ enhances chemo-sensitivity (86) - Treatment: LNPs-miR-182-3p inhibits brain metastases (83)	- High stability in biofluids - Tissue-specific expression - Early dysregulation in cancer - Multiplex detection feasible	- Short length complicates amplification - Low abundance - Batch variation in RT-qPCR - Requires normalization controls
lncRNA	- >200 nt, no protein-coding - Roles: acting as scaffolds, decoys, or guides; - Modulates signaling/chromatin - Dysregulated in TNBC	- Diagnosis: XIST↑, NEAT1↑ in TNBC serum (93); RP11-434D9.1, LINC00052 differentiate TNBC (94) - Prognosis: T376626↑ linked to advanced stage/poor survival (101) LINK-A↑ predicts relapse (102) - Drug resistance: HIF1A-AS2/AK124454 promote taxane resistance (100) - Targets: MILIP knockdown inhibits TNBC growth (96)	- Cancer-specific expression - Stable in serum - Functional relevance in metastasis/drug resistance	- Complex secondary structures - Low abundance in circulation - Lack of standardized detection protocols
Exosome	- 40–160 nm lipid bilayer - Carries molecular cargo - Mediates cellular communication	- Metastasis: Acetylated LAP-TGF-β1 promotes lung metastasis (106) - Diagnosis: Proteomic signatures identify tumor origin (105) - Treatment: Exosome nanoparticles inhibit metastasis (107)	- Molecular shielding - Abundant in biofluids - Mirrors TME	- Isolation technically challenging - Contamination with other vesicles - Standardization issues in profiling
circRNA	- Covalently closed loop - RNase R-resistant - High conservation - Regulates gene expression	- Prognosis: circRNA-CREIT↓ linked to poor prognosis in TNBC (109) - Therapy resistance: circHIF1A↑ promotes proliferation/metastasis (110) - Diagnosis: Differential expression in TNBC vs. non-TNBC (109)	- Exceptional stability - Cancer-specific expression - Resists exonuclease degradation	- Low abundance - Complex detection due to circularity - Limited clinical validation
TANs	- Most abundant immune cells - Promote migration/EMT/ angiogenesis - Form clusters with CTCs	- Prognosis: CTC-neutrophil clusters correlate with poor PFS (114) High TIN density predicts advanced grade/stage (115) - Metastasis: TIMP1 secretion induces EMT (115)	- Reflects TIME - Easily accessible in blood - Treatment dynamics	- Inflammation interference - Isolation complexity - Heterogeneous functional states

cfDNA, Cell-free DNA; CHIP, Clonal Hematopoiesis of Indeterminate Potential; circRNA, Circular RNA; CTCs, Circulating Tumor Cells; ctDNA, Circulating Tumor DNA; DDFS, Distant Disease-Free Survival; EMT, Epithelial-Mesenchymal Transition; lncRNA, Long Non-coding RNA; LNPs, Lipid Nanoparticles; miRNA, MicroRNA; MRD, Minimal Residual Disease; NACT, Neoadjuvant Chemotherapy; nt, Nucleotides; OS, Overall Survival; PFS, Progression-Free Survival; RCB, Residual Cancer Burden; RT-qPCR, Reverse Transcription Quantitative Polymerase Chain Reaction; TANs, Tumor-Associated Neutrophils; TIME, Tumor Immune Microenvironment; TINs, Tumor-Infiltrating Neutrophils; TME, Tumor Microenvironment.

Third, expanding the precision medicine framework requires exploring population-specific biomarker signatures across diverse ethnicities, geographic regions, and age cohorts to address TNBC’s intrinsic molecular heterogeneity. This necessitates merging artificial intelligence with liquid biopsy data to decode complex biomarker patterns while integrating radiomic features from MRI, CT, and PET imaging to quantify tumor burden and spatial heterogeneity.

Finally, accelerating clinical translation demands validating liquid biopsy in prospective trials comparing biopsy-guided interventions versus standard care protocols, developing point-of-care platforms for global accessibility, and establishing international standardization for analytical validation. These foundations will enable liquid biopsy to evolve as an essential companion diagnostic, particularly as novel TNBC therapeutics including immune checkpoint inhibitors, PARP inhibitors, and antibody-drug conjugates enter clinical practice.

Ultimately, the convergence of these advances will transform TNBC management from episodic tissue sampling to continuous molecular monitoring, ushering in an era where real-time liquid biopsy data dynamically navigates precision treatment decisions throughout the patient journey.
